# Multi-scale predictive modeling of phenology and carotenoid content in carrots using spectral techniques, colorimetry, and artificial intelligence

**DOI:** 10.7717/peerj.21389

**Published:** 2026-06-26

**Authors:** Paola Andrea Ospina-Sánchez, Juan Camilo Henao-Rojas, JoaquínGuillermo Ramírez-Gil

**Affiliations:** 1 Facultad de Ciencias Agrarias, Departamento de Agronomía, Laboratorio de Agrocomputación y Análisis Epidemiológico, Universidad Nacional de Colombia, Sede Bogotá, Bogota, Colombia; 2 Centro de Investigación La Selva, Corporación Colombiana de Investigación Agropecuaria (Agrosavia), Rionegro, Colombia

**Keywords:** Non-destructive estimation, Hyperspectral imaging, Machine learning, Deep learning, Predictive modeling, Carrot quality, Precision agriculture

## Abstract

**Objective:**

This study presents an integrated, multi-scale approach for the non-destructive estimation of phenological stages and carotenoid content in carrots by combining spectral techniques, colorimetry, and artificial intelligence.

**Methods:**

Six commercial varieties, including orange, yellow, white, and purple genotypes, were evaluated under field and laboratory conditions using multispectral drone imagery, high-resolution spectroradiometric signatures, red green blue (RGB) images, and CIELAB color measurements. A hierarchical modeling framework was developed across two phases: (i) spectral modeling using uncrewed aerial vehicle (UAV)-based multispectral indices, textural and geometric metrics, and laboratory-generated hyperspectral signatures; and (ii) a colorimetric index from RGB images.

**Results:**

Using UAV-based multispectral field data, phenological prediction indices achieved high classification performance (F1-scores > 0.90) when modeled with a Random Forest classifier, supported by distinct spectral signatures associated with canopy development and senescence. In parallel, carotenoid content estimation using a Random Forest regression model demonstrated strong predictive accuracy (*R*^2^ = 0.897; RMSE = 0.584), with the Plant Senescence Reflectance Index (PSRI) and Carotenoid Reflectance Index (CRI) identified as the most influential predictors. A complementary laboratory-based Random Forest regression model using high-resolution spectral signatures achieved near-perfect predictive performance (*R*^2^ = 0.987). SHapley Additive exPlanations (SHAP) analysis identified physiologically relevant wavelengths in the green (540–550 nm) and red-edge (∼700 nm) regions as the primary drivers of carotenoid concentration. Likewise, a novel colorimetric index (ICarot), derived from CIELAB parameters, enabled accurate image-based carotenoid estimation (*R*^2^ = 0.85).

**Conclusion:**

This study introduces an innovative multi-sensor framework for precision agriculture and automated postharvest quality control, enabling rapid, objective, and scalable phenotyping in carrot production systems. Through the integration of spectral, colorimetric, and AI-based approaches, the proposed methodology effectively captures both internal nutritional attributes and external quality traits within a unified, non-destructive assessment pipeline.

## Introduction

Carotenoids constitute one of the most important groups of plant pigments from nutritional, functional, and physiological perspectives. Synthesized by plants, algae, and some microorganisms, these compounds play essential roles as precursors of vitamin A, natural antioxidants, and protective agents against chronic diseases (Rodriguez-Amaya, 2016). Among horticultural crops, carrot (*Daucus carota* L.) stands out as a primary dietary source of carotenoids, particularly β-carotene and lutein, while pigmented varieties may also contain minor amounts of other carotenoid compounds (Surjadinata, Jacobo-Velázquez & Cisneros-Zevallos, 2021). However, the composition and concentration of these secondary metabolites vary significantly between traditional and unconventional genotypes, posing a persistent challenge for their characterization and for the development of standardized quality assessment methods (Montesano et al., 2020).

These challenges are exacerbated by the low level of technification characterizing the carrot production chain in many regions, where the adoption of precision agriculture tools and automated postharvest sorting systems remains limited (Fuentes, Charnock & Seneweera, 2022). As a consequence, an estimated 20% to 40% of production is lost due to physical damage, diseases, deformities, and failure to meet internal quality standards. Importantly, these losses are not caused solely by biological or mechanical factors themselves, but by the limited availability of rapid, objective, and non-destructive monitoring tools capable of detecting quality deterioration, classifying defects, and supporting timely management decisions. Consequently, the technological gap acts as a key underlying driver that amplifies production losses across the value chain (Kader, 2002; FAO, 2019).

A key factor underlying this variability is the dynamic relationship between carotenoid accumulation and plant phenology. Because pigment content changes throughout the crop cycle in response to genetic, agronomic, and physiological drivers, accurate phenological monitoring is essential for defining optimal harvest timing and predicting final root quality (Gamon et al., 2016). Conventional analytical methods, such as high-performance liquid chromatography (HPLC) provide precise quantification but remain destructive, costly, and impractical for real-time decision-making (Pasquini, 2018). In parallel, external quality assessment commonly relies on subjective visual inspection, limiting reproducibility and contributing to postharvest losses and reduced market competitiveness (Blasco, Cubero & Moltó, 2016; Li, Zhang & Huang, 2020). Together, these constraints highlight the need for rapid, objective, and non-destructive approaches capable of jointly estimating biochemical status, phenological development, and external quality attributes (Zhang, Wang & Li, 2022).

Optical sensing technologies combined with artificial intelligence have emerged as promising alternatives for objective and non-destructive crop assessment. Visible and near-infrared (Vis-NIR) spectroscopy has demonstrated strong potential for predicting bioactive compounds through their characteristic spectral signatures (Cen & He, 2007), while color indices derived from Red, Green, Blue (RGB) imagery enable indirect pigment estimation using color-channel combinations or CIELAB (also known as CIE L*a*b* color space) coordinates with low-cost equipment (Arias, Oria & López-Buesa, 2018; Pathare, Opara & Al-Said, 2013). In carrot production, these approaches have been successfully applied for carotenoid prediction, hyperspectral quality monitoring, and morphology-based classification (Falcioni et al., 2023; Turner et al., 2018), However, most existing studies rely on single sensors, isolated datasets, or single observation scales, limiting comprehensive understanding of the interaction between crop development, pigment dynamics, and final product quality (Weiss et al., 2020).

To address this limitation, the present study proposes a multi-scale, multi-sensor predictive framework integrating uncrewed aerial vehicle (UAV)-derived multispectral imagery, laboratory hyperspectral signatures, RGB-based colorimetric phenotyping, and machine-learning modeling. This integrative strategy enables the simultaneous, non-destructive estimation of phenological stage, carotenoid concentration, and external quality attributes, thereby linking physiological processes across spatial and temporal scales.

Importantly, these methodological components are conceived not as independent analyses but as complementary layers within a unified analytical system, where spectral sensing captures internal biochemical and phenological dynamics, and RGB-based artificial intelligence enables external quality and damage assessment relevant to postharvest decision-making. By coupling heterogeneous optical datasets through artificial intelligence, the framework enhances early detection of quality dynamics, supports harvest decision-making, and facilitates scalable postharvest classification within precision-agriculture systems (Bertin, Génard & Hertog, 2018).

Accordingly, this study aims to develop and validate multi-scale predictive models that integrate multi-source spectral and colorimetric information to improve crop monitoring, quality assessment, and decision-making in carrot production systems. By addressing current limitations in multi-source data integration, the proposed approach provides robust, non-destructive tools for technologically constrained agricultural value chains, ultimately contributing to improved management practices and optimized production performance.

## Materials & Methods

### Description of methodological approach

**Figure 1:**
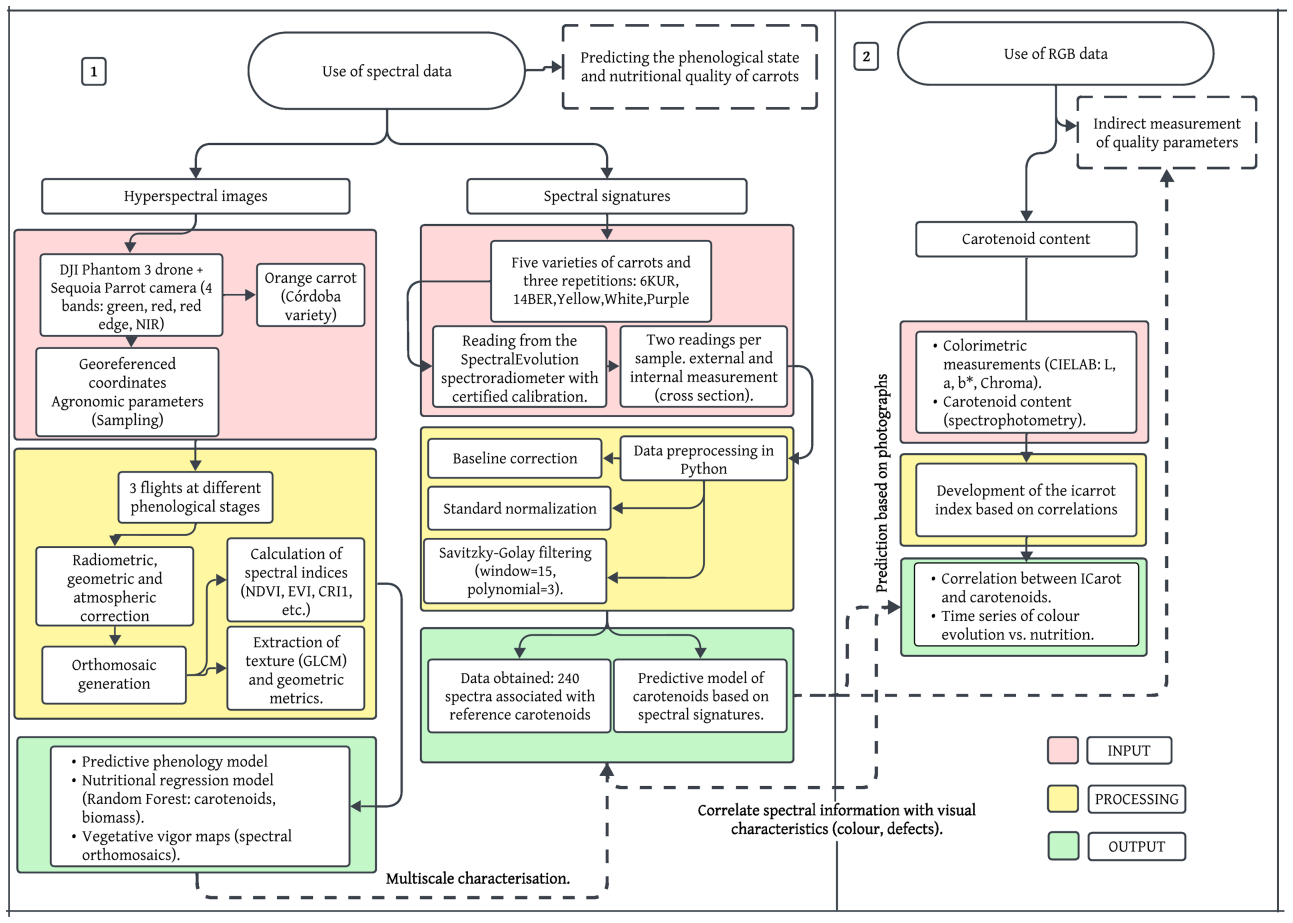
Integrated methodological framework for multi-scale assessment of carrot nutritional quality. Schematic representation of the experimental workflow combining destructive and non-destructive approaches. The framework integrates UAV-based multispectral imagery, laboratory hyperspectral spectral signatures, RGB colorimetric measurements, and physicochemical carotenoid quantification. Data preprocessing, spectral index extraction, machine-learning modeling, and ICarot index development are illustrated, highlighting the multi-scale integration used to predict phenological stage and nutritional quality in carrot crops.

This study followed a two-phase, multi-sensor framework integrating field- and laboratory-scale data for the non-destructive assessment of phenology, nutritional quality, and external damage in carrot crops (*D. carota*), as summarized in Fig. 1. The schematic provides a concise overview linking data sources, preprocessing, predictive modeling, and analytical outputs, while detailed methodological descriptions are presented in the following sections. Phase I focuses on spectral-based prediction of phenological stage and carotenoid content using UAV multispectral imagery and laboratory hyperspectral signatures. Phase II addresses RGB-based phenotyping for carotenoid estimation and automated defect classification through artificial intelligence. Together, both phases establish a coherent multi-scale workflow connecting canopy-level spectral dynamics with root-level nutritional and visual quality.

### Phase I: use of multispectral data images captured by drones to predict phenology and carotenoids in carrots

This first phase integrated multi-source spectral data through a hierarchical modeling framework to enable the indirect prediction of phenological stages, physicochemical properties, and nutritional parameters in carrot crops. The strategy combined drone-based remote sensing and laboratory spectrometry to establish spectral, biophysical relationships across different spatial and temporal scales, thus addressing the challenge of non-destructive crop quality characterization.

### Multispectral data acquisition from cameras attached to drones

This stage aimed to acquire temporally resolved multispectral imagery under actual field conditions to capture spectral dynamics for the indirect assessment of phenological development, physicochemical parameters, and plant vigor.

### Growing conditions for 12NAN variety carrots and sampling for the determination of phenological phases and quality parameters using spectral data

The study was established in a one-hectare plot located in the municipality of Bojacá, Cundinamarca, Colombia (4°38′12.0″N 74°18′02.9″W, 2,600 m a.s.l.), a representative area of the Cundiboyacense high plateau. This region features typical agroecological conditions of the high-altitude Andean zone, characterized by loamy-clay soils and a bimodal rainfall regime with an annual precipitation of approximately 800–1,000 mm (IDEAM, 2023). Soil preparation followed conventional tillage practices, including plowing and harrowing, followed by ridge formation with approximately 0.5 m spacing between rows. Sowing was performed at a density of approximately 1.0–1.2 million seeds ha^−1^.

The crop was managed with a standard base fertilization (N-P-K: 100-60-120 kg/ha), consistent with recommendations for soils in the Andean region (Sánchez, Amezquita & Muñoz, 2021). No supplemental irrigation was applied, allowing the crop to develop under the natural rainfall conditions of the area. The crop was specifically designated for spectral image acquisition using an uncrewed aerial vehicle-UAV (sensors operating at 400–1,000 nm) at different phenological stages. This technique enables the analysis of spectral signatures under real-field conditions, a methodology validated in previous studies (Zhang, Liu & Shang, 2020).

Spectral monitoring of the carrot variety 12NAN was conducted through three strategically timed drone flights aligned with key phenological stages, as defined by the scale for root crops proposed by Meier et al. (2009). The flights were scheduled with a periodicity of approximately 30 to 35 days to capture the transition between these critical physiological phases from active vegetative growth, through peak biomass accumulation, to advanced pre-harvest maturation.

To establish a ground-truth dataset to correlate with spectral image data, a systematic grid sampling protocol was implemented following a standardized methodology for crop monitoring (Daughtry et al., 2006). For each of the three-field sampling period thirty permanent sampling points were georeferenced using a Garmin Global Positioning System (GPS) device and marked within the one-hectare plot. At each point and in each of the three periods, a set of descriptive growth variables was measured non-destructively throughout the crop cycle. Measurements were conducted concurrently with each of the three UAV flights. These flights were scheduled to coincide with key phenological stages of the carrot crop, as follows: the first at 20 days after sowing (DAS), corresponding to principal growth stage 1 (Biologische Bundesanstalt, Bundessortenamt and CHemical industry (BBCH): 19 stage, more than nine true leaves unfolded) (Meier et al., 2009; Kahl, Friedt & Ordon, 2022); the second at 53 DAS, during principal growth stage 4 (BBCH 49 stage, root thickening indicating advanced development of the storage organ) (Meier, 2018; Strehmel, Pérez-de Luque & Maldonado, 2020); and the third at 83 DAS, aligning with principal growth stage 4 (BBCH 47–49 stages, root reaching marketable size and nearing physiological maturity) (Meier, 2018; Daughtry et al., 2006).

The variables assessed at each georeferenced point included leaf length (cm), root diameter (mm), root length (cm), number of leaves per plant, and the respective BBCH phenological stage. This repeated-measures design was considered during model validation to avoid temporal or spatial data leakage.

Upon completion of the crop cycle, representative samples from each sampling point were collected and transported to the laboratory for analysis. The destructive evaluations were conducted following a standardized protocol. Aerial and root biomass were quantified by recording fresh weight using a precision analytical balance (Mettler Toledo XS205, 0.0001g) after blotting excess moisture with absorbent paper. Total soluble solids (TSS, ^∘^Brix) were determined from homogenized pulp using a digital refractometer (HI96821, Hanna Instruments, Inc., Woonsocket, RI, USA) calibrated with distilled water. Total titratable acidity (TTA) was quantified through potentiometric titration using a Metrohm 916 Food Ti-Touch (CH), with a titration endpoint set at pH 8.1 using 0.1N NaOH (Balaguera-López et al., 2017). Total carotenoid content was determined by spectrophotometry following the methodology described by Rodríguez-Amaya & Kimura (2004), using β-carotene as a standard and expressing results as µg β-carotene equivalents per gram of fresh weight. For total dry matter analysis, samples were dried in a forced-air oven at ±70 °C until a constant weight was achieved. All analyses were performed in triplicate, with care taken to avoid adjacent cut surfaces to prevent interference from mechanical damage on subsequent measurements. All data were recorded in traceable spreadsheets that included sample identification, operator name, and timestamp.

### Acquisition and processing of multispectral images data under field conditions

A DJI Phantom 3 Advanced quadcopter equipped with a Parrot Sequoia multispectral camera was used for image acquisition, flown at a constant altitude of 30 m above the crop canopy. The Parrot Sequoia multispectral camera data in four spectral bands: green (530–570 nm), red (640–680 nm), red edge (730–740 nm), and near-infrared (770–810 nm), supplemented by RGB images for visual reference. Each spectral band had a resolution of 1.2 megapixels with 16-bit radiometric depth, enabling detection of subtle variations in leaf reflectance associated with photosynthetic pigment changes. An ambient light sensor continuously recorded solar irradiance during flights, a critical parameter for subsequent radiometric calibration.

The calibration protocol involved imaging a Spectralon reference panel with a known reflectance factor (0.99 within the VIS-NIR range), which served as the standard for converting at-sensor radiance to surface reflectance values. This conversion was performed using an empirical line calibration algorithm to convert at-sensor radiance to surface reflectance. Before this transformation, the raw images underwent radiometric and geometric corrections through specialized Python scripts optimized for the specific camera model, applying dark frame and flat-field calibration parameters to correct for sensor noise and non-uniform pixel response.

Orthomosaic generation was conducted using Agisoft Metashape Professional software (Version 2.0™). The photo alignment process was executed with ‘High’ accuracy, the ’Reference Precision’ parameter was enabled, and noisy point filtering was applied using a reprojection error threshold of 0.3 pixels. Dense cloud construction was generated with ‘High’ quality and a ‘Moderate’ depth filtering mode. For the digital surface model and orthomosaic generation, the interpolation method was set to ‘Enabled’, with a cell size corresponding to the target spatial resolution. Six strategically distributed ground control points, surveyed with a differential GPS, were used to ensure geometric accuracy during the scene alignment and optimization process. Each resulting orthomosaic exhibited a uniform spatial resolution of 2.67 cm/pixel, consistent band dimensions, and precise spatial co-registration across all spectral bands.

The flight schedule was carefully designed to capture spectral dynamics linked to key physiological processes. The initial flight coincided with Stage 3 (rosette growth: 6–8 true leaves) (Meier et al., 2009), corresponding to peak vegetative growth (Gitelson, Gritz & Merzlyak, 2003). The second flight was conducted during Stage 4 (root thickening) to monitor maximum biomass and pigment accumulation (Sims & Gamon, 2002). The final flight was scheduled at Stage 5 (maturity) to target the commercial maturity phase, when carotenoid levels peak (Blackburn, 2007). This temporal sampling scheme enabled characterization of carotenoid spectral evolution, particularly through analysis of the red-edge band (730–740 nm), known for its sensitivity to these secondary compounds (Gitelson, Keydan & Merzlyak, 2006).

The number and timing of UAV flights were determined based on the need to capture the principal physiological transitions of carrot development according to the BBCH scale, ensuring representation of early vegetative growth, advanced root thickening, and pre-harvest physiological maturity. These stages correspond to periods of major variation in pigment accumulation and canopy spectral response reported in previous studies, thereby providing a biologically meaningful temporal framework for spectral–phenological modeling rather than an arbitrary sampling schedule.

### Determination of spectral, textural, and geometric indices as predictive variables

A multi-faceted analytical approach was employed to quantify the crop’s physiological and biochemical status. This included the calculation of vegetation indices, textural features, and geometric metrics derived from the processed orthomosaics.

Vegetation indices were computed from algebraic combinations of spectral bands to serve as proxies for key agronomic traits such as biomass, chlorophyll content, and plant vigor (Thenkabail, Lyon & Huete, 2019; Xue & Su, 2017). A comprehensive list of the indices calculated is provided in Table S1. In addition, textural features were extracted to quantify the spatial heterogeneity of the canopy, which is influenced by plant architecture and leaf distribution. These metrics were based on the Grey-Level Co-Occurrence Matrix (GLCM) (Haralick, Shanmugam & Dinstein, 1973) applied to the NIR band due to its sensitivity to canopy structure and pigment distribution. Calculations were performed for distances of 1, 3, and 5 pixels and angles of 0° and 45°. The specific texture properties analyzed (*e.g.*, contrast, entropy) are detailed in Table S2. On the other hand, Geometric metrics were obtained to describe the morphology and size distribution of canopy objects. These properties were derived from binary images generated using the Otsu (1979) thresholding method, with connected components analyzed following the principles of Vincent & Soille (1991), as implemented by Russ & Neal (2016). The full set of geometric features extracted is enumerated in Table S3.

For each calculated index and metric (spectral, textural, and geometric), a set of seven descriptive statistics consisting of mean, median, standard deviation, 10% quantile, 90% quantile, minimum, and maximum value was extracted to characterize the central tendency and variability within the experimental plots.

### Predictive modeling of phenological status and quality parameters (carotenoids) using spectral data acquired in the field

Two independent predictive models were developed to address different aspects of crop monitoring: (1) a classification model for plant phenology and (2) a regression model for physicochemical parameters. Both models utilized six spectral indices as predictor variables: Normalized Difference Vegetation Index (NDVI), Green Normalized Difference Vegetation Index (GNDVI), Visible Atmospherically Resistant Index (VARI), Normalized Difference Red-Edge Index (NDRE), Carotenoid Reflectance Index (CRI), and Plant Senescence Reflectance Index (PSRI), derived from multispectral imagery with a spatial resolution of 2.67 cm/pixel. These features were linked to corresponding field measurements from each monitored plot through precise georeferenced coordinates and sampling dates.

Phenological Classification Model: A phenological classification model was developed to predict crop development stages using spectral indices as predictor variables. The Random Forest algorithm was selected following a comparative evaluation against alternatives such as eXtreme Gradient Boosting (XGBoost) and Support Vector Machines, demonstrating an optimal balance between predictive performance and stability on moderately sized datasets. This performance advantage is attributed to its capacity to model the complex nonlinear relationships present in phenological data while maintaining lower prediction variance than more complex boosting algorithms.

The specific classifier configuration employed 120 estimators with maximum depth limited to three and a minimum of three samples per leaf, parameters optimized to prevent overfitting while capturing underlying data relationships. The mapping of dates to phenological categories followed established agronomic criteria, replicating expected physiological transitions in the crop based on field morphological measurements that included root length and diameter, leaf length, and foliar count. (i) Stage 0 (Germination and Emergence): 20 DAS, BBCH 00-09. Characterized by first true leaves and initial root development, with seedlings reaching 5–10 cm in height (Meier, 2018; Rubatzky, Quiros & Simon, 1999). (ii) Stage 1 (Vegetative Growth): 50 DAS, BBCH 10-39. Identified by 8–12 true leaves, root thickening, and 40–60% ground cover (Brewster, 2008; Meier, 2018). (iii) Stage 2 (Root Maturation): 80 DAS, BBCH 40-49. Defined by the attainment of commercial root diameter (1.5–2.5 cm), development of orange pigmentation, and active carotenoid accumulation (Simon, Peterson & Gabelman, 1980).

Model evaluation implemented stratified 10-fold cross-validation with random seed 42, ensuring each observation was used exactly once for validation while preserving proportional class distribution in each partition. The classification report included precision, recall, and weighted F1-score to account for the multi-class distribution, complemented by a confusion matrix to visualize error patterns between consecutive phenological stages.

To ensure methodological transparency and to avoid potential bias derived from repeated spatio-temporal measurements, the distribution of observations across phenological stages was explicitly balanced. Each phenological class was represented by measurements collected from the same set of 30 georeferenced sampling points across the three UAV acquisition dates, guaranteeing comparable sample sizes and consistent spatial representation.

Because the dataset contained repeated observations over time from identical spatial locations, special care was taken to prevent temporal autocorrelation and data leakage during model validation. Cross-validation was therefore implemented using a stratified scheme constrained simultaneously by sampling date and georeferenced point, ensuring that observations originating from the same UAV flight or spatial position were not included in both training and validation subsets. This strategy preserved the statistical independence of validation data and prevented the model from learning temporal or spatial identifiers instead of biologically meaningful spectral relationships.

**Figure 2:**
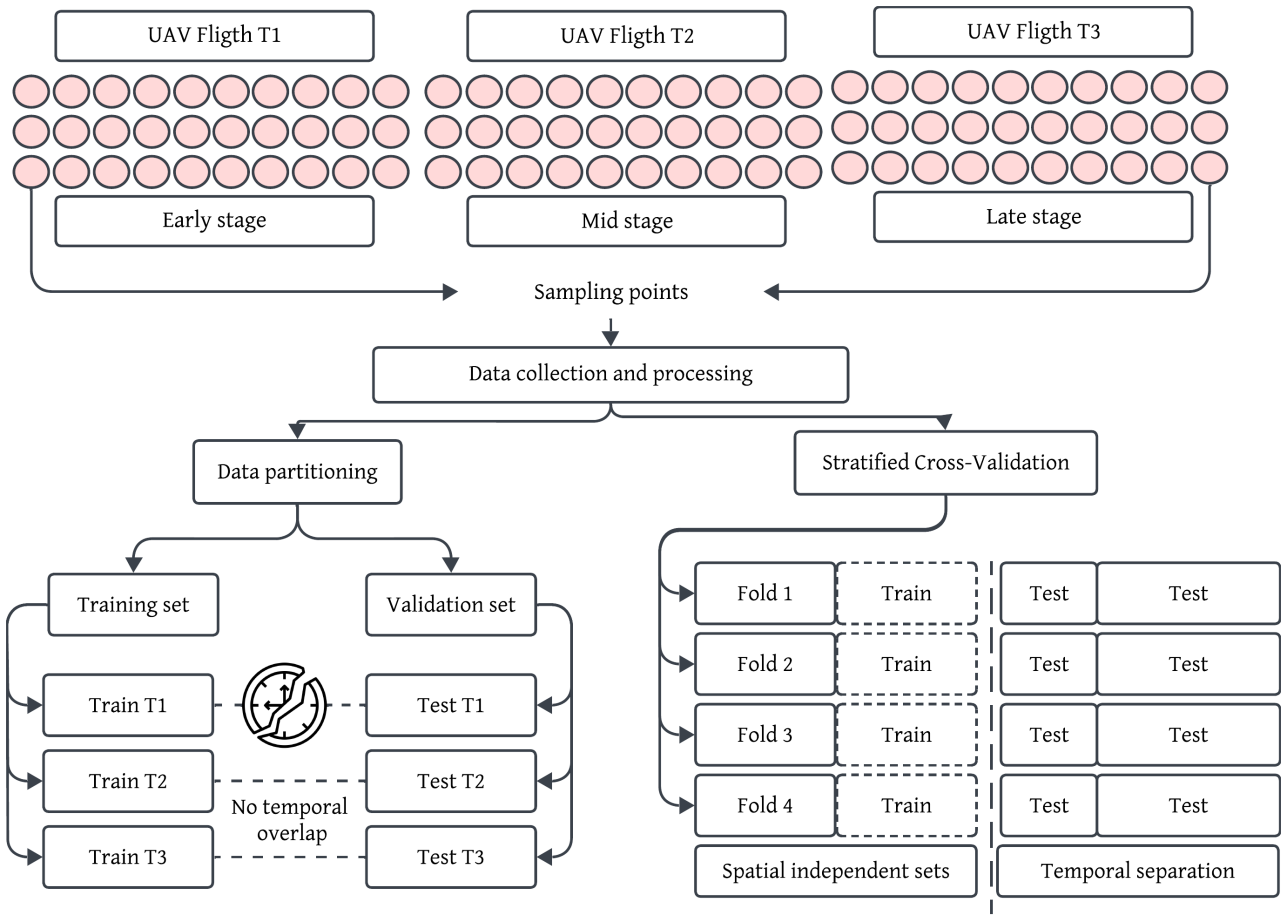
Data partitioning and validation strategy for UAV-derived spectral modeling. Schematic representation of the temporal and spatial data separation procedure used to prevent autocorrelation and data leakage. Observations collected from three UAV flights across 30 georeferenced sampling points were partitioned using stratified cross-validation constrained by sampling date and spatial location, ensuring independence between training and validation subsets and balanced representation of phenological stages.

A schematic representation of the complete data partitioning and validation workflow is provided in Fig. 2 to enhance reproducibility and facilitate interpretation of the machine-learning framework.

Carotenoid regression model: The objective of this model was to develop and validate a multivariate nonlinear regression model to accurately estimate total carotenoid content in the crop, using exclusively spectral data derived from multispectral imagery. The target variable consisted of carotenoid concentrations determined through standard spectrophotometry, providing a reliable reference dataset for model development.

The selection of the Random Forest Regressor algorithm was based on its inherent capacity to handle complex nonlinear relationships between predictor variables, a critical characteristic given that interactions between spectral indices do not necessarily follow linear patterns. This algorithm demonstrated superior performance in capturing variable interaction effects, where the ensemble structure of Random Forest exhibited greater capacity to model complex interdependencies between spectral bands without requiring complex input data transformations. The model configuration employed 300 estimators with a maximum depth of five and a minimum of two samples per leaf, parameters optimized through cross-validation to balance predictive capability and generalization. The overfitting prevention strategy incorporated multiple techniques, including bootstrap aggregation, limited tree depth, and minimum sample requirements for node splitting, thereby ensuring final model robustness.

The evaluation process implemented 10-fold cross-validation with comprehensive metrics including Coefficient of determination (R^2^) for explained variance, Root Mean Square Error (RMSE) for error in original units and Mean Absolute Error (MAE) for direct interpretability. Additionally, SHapley Additive exPlanations (SHAP) analysis was implemented to disaggregate contributions of each spectral index in individual predictions, identifying CRI and PSRI as dominant predictors due to their specific spectral sensitivity to carotenoid pigments, while NDVI and GNDVI provided complementary information about overall vegetation status.

Spatio-temporal integration of UAV-derived data with spectrophotometric measurements was ensured through georeferenced coordinates and sampling dates, establishing precise one-to-one correspondence between spectral patterns and measured carotenoid concentrations. Specifically, thirty permanent georeferenced sampling points distributed across the experimental plot were monitored during three UAV acquisition dates corresponding to key phenological stages, generating repeated spectral observations for each spatial location.

This structured sampling scheme provided the spectral dataset used for machine-learning training and validation, ensuring consistent spatial representation and temporal comparability across phenological classes. All processes implemented random_state = 42 to guarantee exact reproducibility in future replications, while feature importance analyses provided physiological validation for identified relationships between spectral signatures and biochemical contents.

### Indirect determination of quality characteristics (carotenoids) in carrots using hyperspectral data acquired by a fixed spectroradiometer

This stage aimed to establish fundamental spectral pigment relationships through controlled laboratory measurements to enable indirect estimation of carotenoid content, while implementing explainable AI techniques to identify spectral regions and interpret relationships between spectral signatures and carotenoid patterns.

### Growing conditions for carrots sown in Agrosavia

This section utilized carrot plants (*D. carota*) from five distinct genotypes: 14BER, 6KUR, white, purple, and yellow. These specific varieties were selected for their commercial prevalence and common cultivation practices in the department of Antioquia, Colombia. The field experiment was established under a randomized complete block design (RCBD) with four replicates, in which each genotype was randomly assigned to experimental plots within each block. This design allowed control of spatial field variability and ensured robust comparisons among genotypes. The cultivation was conducted in open-field conditions at the La Selva Research Center of AGROSAVIA (6°08′06″N, 75°25′03″W; 2,120 m.a.s.l.). The site is classified within the lower montane moist forest (bh-MB) life zone, characterized by an average annual temperature of 17 °C and 78% relative humidity. All agronomic management, including fertilization, irrigation, and pest control, adhered to standardized protocols as defined by the project’s agronomic team to ensure consistency. Seeds were manually sown on cultivation beds (1.20 m × 5.00 m × 0.30 m) at a depth of 0.5 cm, with a spacing of 10 cm between plants and 15 cm between rows. Upon reaching physiological maturity, carrots were harvested from a central 2 m^2^ area within each bed to exclude border effects and reduce variability from inter-plant competition. A total of 120 carrot roots (corresponding to 24 samples per genotype across the five evaluated varieties) were harvested. Upon harvest, the roots were washed, and any damaged samples were discarded. They were then transported fresh under refrigerated conditions to the laboratories at the Universidad Nacional de Colombia, Bogotá campus.

The target variable is the total carotenoid content, expressed in parts per million (ppm). This reference data was obtained for each carrot sample using standard spectrophotometric techniques following the protocol described by Horwitz (2002). Briefly, carotenoids were extracted from a homogeneous tissue sample using acetone, and the concentration was quantified by measuring absorbance at a specific wavelength (450 nm) using a calibration curve with β-carotene as a standard. This resulted in a precise ppm value for each of the 120 samples, which served as the ground truth for model training and validation.

### Evaluation of the spectral response of carrot genotypes using a fixed spectradiometer

Hyperspectral measurements were conducted under controlled laboratory conditions using a portable SM-1900 spectroradiometer (Spectral Evolution, Haverhill, MA, USA), following a protocol adapted from established methodologies for plant pigment analysis (*e.g.*, Blackburn, 2007; Ustin et al., 2009). To ensure measurement accuracy and reproducibility, a dark chamber lined with matte black material was used to eliminate ambient light and minimize unwanted reflections, as per the manufacturer’s guidelines and standard laboratory practice. Lighting was controlled using an integrated halogen source (20W, 2856K), with a measurement geometry of 90°/25° (incidence angle/field of view) and a constant distance of 15 cm between the sensor and sample. The equipment provided a spectral resolution of 3.5 nm (350–1,000 nm) and 10 nm (1,000–1,900 nm), enabling the detailed capture of spectral signatures associated with carotenoids and other pigments.

Each carrot sample (constituting one biological replicate per variety) was analyzed weekly over a period of four weeks to monitor temporal changes in spectral signatures. Measurements were conducted at weekly intervals (Week 1, Week 2, Week 3, and Week 4) on both the external surface and an internal cross-section of the sample, generating two reflectance spectra in .sed format per sample per week. This process was repeated for three independent biological replicates per variety (*i.e.,* three individual carrots from different plants for each of the five varieties: Yellow, Purple, White, 6KUR, and 14BER), resulting in a total of 120 raw spectral data files (five varieties × 3 replicates × 2 surfaces × 4 weeks).

Data processing was performed through a custom Python pipeline that included baseline correction, Savitzky-Golay filtering for spectral smoothing and noise reduction (Savitzky & Golay, 1964), and standard normalization (SNV). For the subsequent modeling, the spectra from the internal and external measurements for each biological replicate at each time point were averaged to produce a single, representative spectral signature per individual carrot per week. Consequently, the final validated dataset integrated 120 averaged spectra, which served as the predictor variables (features) for the model. Each of these observations was associated with its corresponding reference carotenoid value (obtained through standard spectrophotometric techniques; Horwitz, 2002) from the same individual carrot at the same weekly time point, serving as the target variable for prediction.

### Carotenoid predictive modeling using hyperspectral signatures

This subsection aimed to develop a highly accurate predictive model that estimates the concentration of carotenoids in plant tissue solely from its spectral signature. The ultimate goal was to create a tool where inputting a high-resolution spectral reflectance curve, obtained *via* a spectroradiometer, would directly output a precise quantitative prediction of carotenoid content, thereby enabling rapid, non-destructive assessment.

The quantification of β-carotene content was performed following a protocol facilitated by the Lab. Química de Productos Naturales of AGROSAVIA- La Selva Research Center of AGROSAVIA (6°08′06″N, 75°25′03″W; 2,120 m.a.s.l.). Initially, standard solutions were prepared from a certified reference material (Merck, Rahway, NJ, USA). A precise amount of 5.0 mg (±0.1 mg) of β-carotene was weighed into a five mL volumetric flask, which was then filled to volume with HPLC-grade acetone and homogenized using a vortex mixer for 10 min. To prevent photodegradation, the process was carried out under light-protected conditions. From this stock solution, working standards of 100 mg/L, 10 mg/L, and 1 mg/L were prepared through successive dilutions. Aliquots were taken with a micropipette, transferred to 10 mL volumetric flasks, and brought to volume with HPLC-grade acetone, followed by an additional 10-minute vortex homogenization.

A calibration curve was constructed using defined aliquots of each standard diluted in a methanol/acetone mixture (50:50), vortexed, and stored in amber vials prior to spectrophotometric analysis This analytical protocol was applied within a structured experimental design to quantify carotenoid content across carrot varieties and post-harvest storage time. The study considered two main factors: carrot variety (five genotypes) and storage time (four weekly intervals), with the experimental unit defined as an individual carrot root Three independent biological replicates were analyzed per treatment combination, resulting in a total of 60 observations (5 varieties × 4 weeks × 3 replicates). Each observation generated a carotenoid concentration value (ppm) used as ground-truth data for predictive modeling.

The Random Forest (RF) algorithm was selected for predictive modeling due to its well-established ability to handle complex nonlinear relationships and its inherent robustness against multicollinearity, a common characteristic of hyperspectral data (Breiman, 2001).

The final model architecture comprised 300 decision trees, a maximum depth of 12 levels per tree, and a minimum of five samples required to split a leaf node. This was not predetermined; instead being the product of a hyperparameter tuning process. We performed a grid search (Bergstra & Bengio, 2012), optimized through variety-stratified k-fold cross-validation (Kuhn & Johnson, 2013), to ensure generalizability and balanced performance across all genotypes. This process consisted of the systematic evaluation of a range of values for the number of trees, maximum depth, and minimum leaf samples. The combination of 300 trees, a max depth of 12, and min five samples per leaf was identified as the optimal configuration, providing the best trade-off between model performance (maximizing predictive accuracy) and computational efficiency (avoiding overfitting from excessive complexity).

To move beyond standard feature importance metrics and gain a deeper, more robust understanding of the model’s predictions, we performed a SHAP analysis (Lundberg & Lee, 2017). This game-theoretic approach quantifies the marginal contribution of each wavelength (feature) to the final prediction for every individual observation (Lundberg & Lee, 2017). We employed SHAP for two primary reasons: (1) its consistency properties ensure that features are accurately ranked by their impact (Lundberg et al., 2020), and (2) it allows for the interpretation of both global model behavior (in which features are most important overall) and local explanations (how features contributed to a specific prediction for a single carrot sample) (Molnar, 2022; Lundberg & Lee, 2017).

To evaluate the potential risk of overfitting associated with the high predictive performance of the hyperspectral regression model, an additional validation analysis was conducted within the cross-validation framework. Model complexity was constrained through hyperparameter tuning, limiting tree depth and enforcing minimum leaf sample requirements, while bootstrap aggregation inherent to the Random Forest algorithm further reduced variance and improved generalization.

Predictive performance remained stable between training and validation partitions, with no substantial degradation in R^2^, RMSE, or MAE across folds, indicating that the model did not memorize training observations but instead captured consistent spectral–biochemical relationships. This stability supports the robustness and generalization capacity of the hyperspectral model.

Furthermore, SHAP analysis identified dominant wavelengths located in physiologically meaningful spectral regions associated with carotenoid absorption and chlorophyll interaction, providing independent biological validation that the model learned interpretable biochemical patterns rather than noise-driven correlations.

### Phase II: Use of RGB data for the determination of carotenoid content in carrots

This second phase aimed to develop artificial intelligence tools that extract quantitative phenotypic information from RGB imagery, enabling indirect prediction of nutritional content through colorimetric analysis and automated classification of physiological defects using deep learning architectures.

### RGB image acquisition and processing

RGB images of the five carrot varieties (6KUR, 14BER, white, purple, and yellow) were systematically captured throughout the experimental period to document morphological development and sample condition over time. Photographs were taken during weekly sampling sessions, in which multiple representative roots per variety were imaged rather than a fixed number of three samples, with a specific focus on capturing images under the most contrasting lighting conditions available: under direct artificial light in the laboratory and under indirect natural daylight. This approach was designed to ensure the model’s robustness to variations in illumination.

The image acquisition was performed using a variety of consumer-grade mobile devices to introduce diversity in sensor characteristics and image processing algorithms into the dataset. The specific devices employed were an Apple iPhone 13, a Samsung Galaxy S21, and a Huawei MatePad 10.4 tablet. The primary camera hardware specifications for these devices are as follows: the iPhone 13 features a 12 MP sensor with an f/1.6 aperture, the Samsung Galaxy S21 utilizes a 12 MP sensor with an f/1.8 aperture, and the Huawei MatePad 10.4 is equipped with an 8 MP sensor with an f/1.8 aperture. Not all carrot roots were photographed with all devices; instead, a representative subset of samples was captured across devices while maintaining identical acquisition geometry, neutral background, camera-to-sample distance, and illumination conditions for all analytical images. All photographs were taken in a controlled manner, ensuring each carrot sample was centered, in focus, and captured against a neutral background under consistent lighting conditions to minimize distractions and isolate the consistent visual features of the carrots themselves.

The criteria for selecting which photographs to take and include in the final dataset were based on clarity, focus, and the accurate representation of the carrot’s phenotypic traits (*e.g.*, color, shape, size). Blurry, out-of-focus, or poorly framed images were discarded. Although acquisition was conducted under controlled focus conditions, a post-acquisition quality-control filtering step based on image sharpness metrics (Laplacian variance threshold) was applied to objectively exclude occasional blurred images, thereby resolving potential inconsistencies between controlled capture and dataset curation. Subsequently, all images were organized into a structured dataset with subdirectories corresponding to each target variety. To address the limited initial number of photographs and to prevent overfitting, a data augmentation strategy was employed. The dataset was artificially expanded by applying a series of random transformations to each original image, including rotations (within a 0–45 degree range), horizontal and vertical flips, and slight zoom variations. This generated new, unique training instances from the existing data.

Following augmentation, all images were standardized to ensure uniform input for the neural network. Each photograph was first cropped to a consistent 5:7 aspect ratio using the Windows cropping tool to maintain uniform composition. Finally, all images were resized to a fixed dimension of 200  ×  200 pixels and normalized by dividing each pixel value by 255, scaling the data to the [0, 1] range. This preprocessing step was critical for data standardization prior to model training. This collection of images was used to build dataset for carotenoid prediction: Images of healthy samples in optimal condition were used, associating each image with the reference carotenoid value of its corresponding sample.

### Development and validation of the ICarot index using colorimetry and RGB data

This stage involved the design and validation of the ICarot index to enable indirect prediction of carotenoid content through color space transformations, establishing a quantitative relationship between CIELAB color coordinates and nutritional parameters in carrot varieties. The predictor variables for the index are the color coordinates in the CIELAB space (L, a, b*). To obtain highly accurate and standardized reference values for development and validation, color was quantitatively assessed for each of the carrot samples using a Konica Minolta CR-400 colorimeter. Measurements were taken at three equidistant points along the central axis of each carrot root, accounting for natural color variability. The colorimeter was calibrated with a standard white tile before each measurement session. All measurements were performed under a D65 illuminant to simulate daylight and at a 10° viewing angle, with results expressed in the CIELAB color space. The average of the three measurements per sample was calculated and used for subsequent analysis. This provided a precise ground truth dataset of color values directly correlated with each sample.

The ICarot index is grounded in the scientifically established relationship between carotenoid pigments (*e.g.*, β-carotene) and color coordinates in the CIELAB space. Specifically, the b* parameter (yellow-blue axis) demonstrates strong positive correlation with carotenoid concentration (Pathare, Opara & Al-Said, 2013), while the a* component (red-green axis) captures the influence of secondary pigments. Chroma (color saturation) reflects pigment purity and intensity (León et al., 2006), with elevated b* and chroma values corresponding to higher carotenoid levels (Meléndez-Martínez et al., 2019).

The selection of the CIELAB parameters a*, b*, and chroma was guided by both physiological relevance and statistical independence in relation to carotenoid variability. The b* coordinate is widely recognized as the primary chromatic descriptor associated with yellow–orange pigmentation and therefore with carotenoid concentration, while a* captures secondary red–green tonal variation linked to pigment interactions and cultivar-specific coloration. Although chroma is mathematically derived from a* and b*, it represents color saturation intensity, a perceptual dimension not fully explained by the linear contribution of the individual coordinates. Multicollinearity diagnostics performed during model calibration confirmed that chroma contributed additional explanatory variance and improved predictive stability compared with models based solely on a* and b*.

The innovation of the ICarot index lies in its weighted formulation, which is designed to optimize the combination of parameters to maximize correlation with ground-truth reference data. Unlike traditional indices based on simple ratios *e.g.*, *a/b*, (*a/b*)^2^, or *b* alone), ICarot integrates three CIELAB derived components, *b*, *a*, and Chroma (*C*), through a balanced weighting system: (70%), (20%), and (10%), respectively. These weights were derived from a multiple linear regression (MLR) analysis performed on the calibration dataset, in which carotenoid concentration served as the dependent variable and b, a, and C as independent predictors. Standardized regression coefficients (β), representing the relative contribution of each predictor, provided the statistical basis for weight assignment. This data-driven approach, supported by established statistical principles (Wu & Sun, 2013) ensures that the index reflects the underlying pigment dynamics and demonstrates superior explanatory performance compared with individual predictors or non-weighted combinations.

Furthermore, the index incorporates a normalization procedure based on study-specific maximum values for each parameter (*b*_*m*__*a*__*x*_, *a*_*m*__*a*__*x*_, *C*_*m*__*a*__*x*_). These maximum values were defined as the 95th percentile of the data distribution for each variable within the calibration dataset. This strategy (i) reduces the influence of extreme outliers that could distort scaling and (ii) stabilizes variance among weighted components, ensuring balanced contribution across predictors. In the model, these constants serve as scaling factors that transform the raw values from individual samples onto a relative, dimensionless scale. To further substantiate the statistical validity of the index, a comparative validation and sensitivity analysis was conducted against commonly used color descriptors and indices, including a/b, Hue angle, and Chroma. ICarot achieved significantly stronger correlations (*p* < 0.05) with reference carotenoid concentrations and lower prediction errors than simpler descriptors. Sensitivity testing based on systematic perturbations of ±10% in the assigned weights confirmed that the original 70/20/10 configuration provided the optimal balance between predictive accuracy (R^2^) and error minimization while remaining stable under small parameter variations. These results confirm the robustness, statistical superiority, and practical reliability of ICarot as a low-cost, non-destructive estimator of carotenoid content.

Beyond quantitative carotenoid estimation, ICarot enables sample classification into three levels (low, medium, high) using percentile thresholds. This classification was validated through visualizations generated with seaborn and matplotlib libraries, confirming alignment with spectroscopic data and machine learning predictions (Waskom, 2021; Hunter, 2007).

The ICarot index is calculated based on Eq. (1).

(1) \begin{eqnarray*}ICarot=0.7\times \frac{{b}^{\ast }}{{b}_{max}^{\ast }} +0.2\times \frac{{a}^{\ast }}{{a}_{max}^{\ast }} +0.1\times \frac{C}{{C}_{max}} \end{eqnarray*}

b* = Yellow-blue axis, associated with carotenoid content (*e.g.*, β-carotene).

a* = Red-green axis, representing the contribution of secondary pigments.

C = Color saturation, related to pigment purity.

b*max,a*max, cmax = Maximum values for each respective parameter obtained from the entire sample population under study.

Validation was performed using colorimeter values (CIELAB color space), and the carotenoid values obtained by spectrophotometry were associated with the image of each sample.

Once this correlation was established and validated against the reference method, the ICarot index was implemented into a computational pipeline for practical, non-destructive evaluation using digital images (Aguilar, García & Martínez, 2023). The implementation process begins with the acquisition and preprocessing of a digital image of the carrot sample, which is resized to standard dimensions to ensure consistent processing (Gonzalez & Woods, 2018). The image is converted from the RGB color space (native to OpenCV) to the CIELAB color space, providing the perceptually uniform color coordinates required for the index (Sharma, 2003). The average values of the L, a, and b* channels are subsequently extracted and scaled, and the validated ICarot formula is applied, calculating Chroma dynamically for each image (León et al., 2006). The calculated index value is finally used to predict the carotenoid content in parts per million (ppm) and to classify the sample into a qualitative category (Low, Medium, High) based on empirically determined thresholds.

## Results

### Phase I: Use of spectral data (multispectral and hyperspectral) for determining carrot phenology and quality under field and laboratory conditions

#### Dynamics of spectral indices of carrot under field conditions

**Figure 3:**
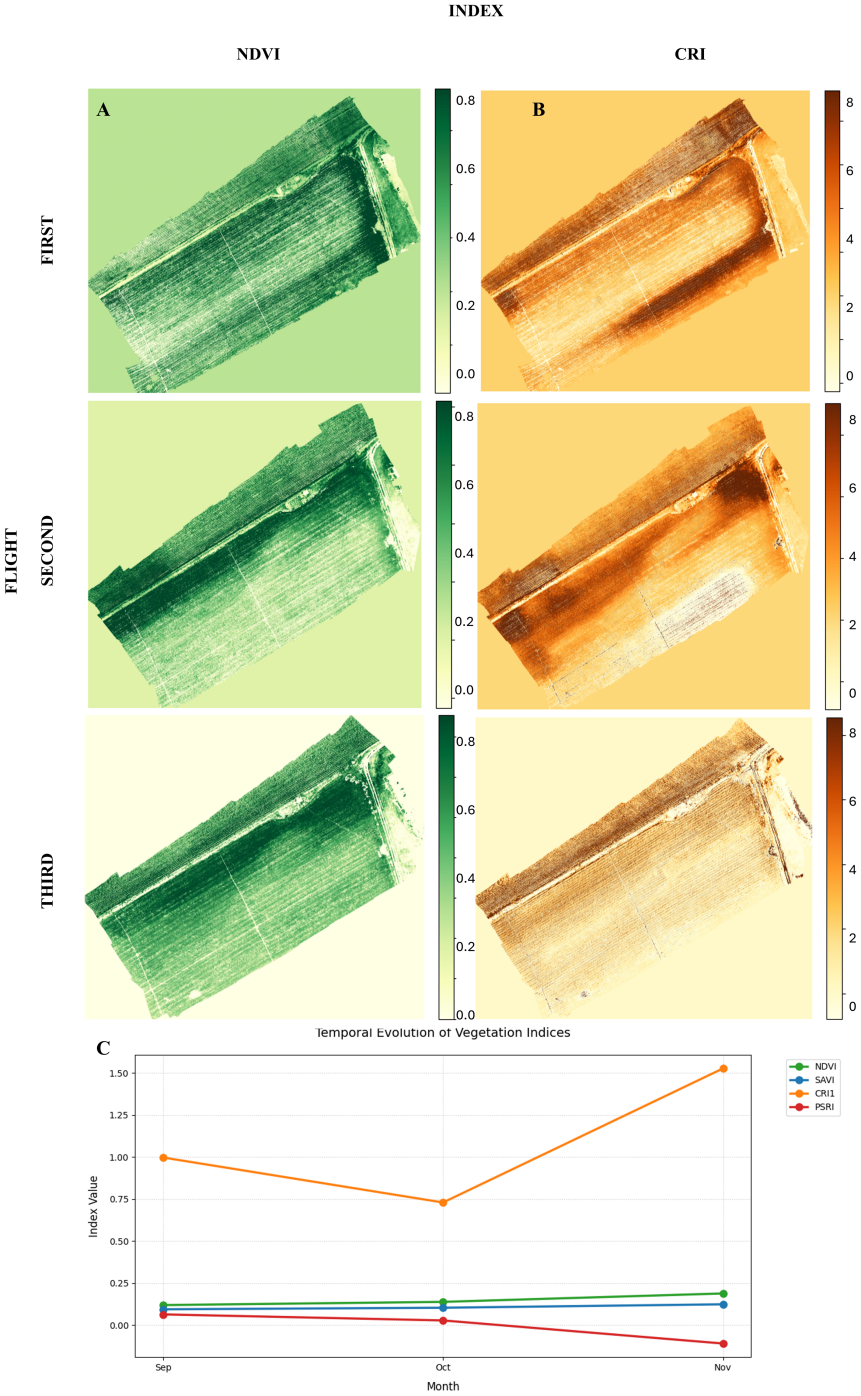
Temporal dynamics of UAV-derived vegetation indices across the crop cycle. Spatial distribution maps of vegetation indices calculated from multispectral UAV imagery at three phenological time points. (A) NDVI (Normalized Difference Vegetation Index), (B) SAVI (Soil-Adjusted Vegetation Index), (C) CRI1 (Carotenoid Reflectance Index 1), and (D) PSRI (Plant Senescence Reflectance Index). (E) Temporal evolution of mean index values across sampling dates. Together, these indices describe changes in canopy vigor, pigment composition, and senescence processes during crop development.

Analysis of the orthomosaics revealed a clear and consistent trajectory of textural and spectral evolution, corresponding to distinct phenological stages of the crop canopy. The textural metrics delineated a clear progression in surface structure, beginning with a heterogeneous and fragmented canopy structure in its early developmental stage, characterized by high contrast and dissimilarity alongside low solidity. A subsequent shift towards a more uniform and consolidated texture was observed, marked by a significant decrease in contrast and a notable increase in correlation and solidity, indicating a period of canopy consolidation and peak vegetative growth. In the final stage, a significant expansion in area was accompanied by a return to higher contrast, coupled with the highest solidity value, suggesting a phase of canopy expansion and maturation where structures became larger and more defined, yet more heterogeneous due to the onset of senescence (Fig. 3 and Fig. S1).

Spectral indices provided a physiological explanation for these structural changes. NDVI values were initially low (predominantly 0.2–0.4), consistent with early growth stages, before a significant increase during the intermediate period (0.2–0.7) that reflected active vegetative growth and increasing biomass. Values were sustained at an elevated level in the final period but with a slight decline, suggesting the initial onset of stress or senescence. The SAVI index, which minimizes soil background effects, strongly mirrored the NDVI trend, confirming the reliability of the observed photosynthetic activity dynamics. The dynamics of the CRI1 further elucidated the pigment composition underlying these phases. Low initial CRI1 values were followed by a pronounced peak synchronized with the maximum NDVI and SAVI, indicating active accumulation of photoprotective carotenoids alongside chlorophyll during the peak of vegetative vigor. The subsequent decline in CRI1 during the final stage, aligning with the reduction in the primary photosynthetic indices, supports the hypothesis of functional pigment degradation during senescence (Fig. 3 and Fig. S1).

Conversely, the PSRI, a specific indicator of senescence, exhibited a robust inverse relationship. Its values were lowest during the peak of photosynthetic activity and showed a pronounced increase in the final stage, directly corresponding to the declines in NDVI, SAVI, and CRI1, thus providing direct spectral evidence of advancing senescence, likely marked by the accumulation of chlorophyll degradation products (Fig. 3 and Fig. S1).

The strength and validity of these physiological relationships were confirmed statistically. A strong positive correlation was found between NDVI, SAVI, and CRI1 during the growth phase (*e.g.*, Pearson *r* > 0.90 between NDVI and CRI1), confirming their shared sensitivity to green biomass and photosynthetic capacity. A linear regression model indicated that NDVI explained a large proportion of the variability in CRI1 (*R*^2^ = 0.87), underscoring their close physiological linkage. All these correlations were highly significant (*p* < 0.001). Simultaneously, a robust and consistently significant inverse correlation of PSRI with all three indices throughout the observation period (*r* < −0.88, *p* < 0.001) statistically confirms its role as a clear indicator of physiological decline, effectively marking the transition from growth to senescence.

#### Phenological classification of carrot model based on field multispectral image data

**Figure 4:**
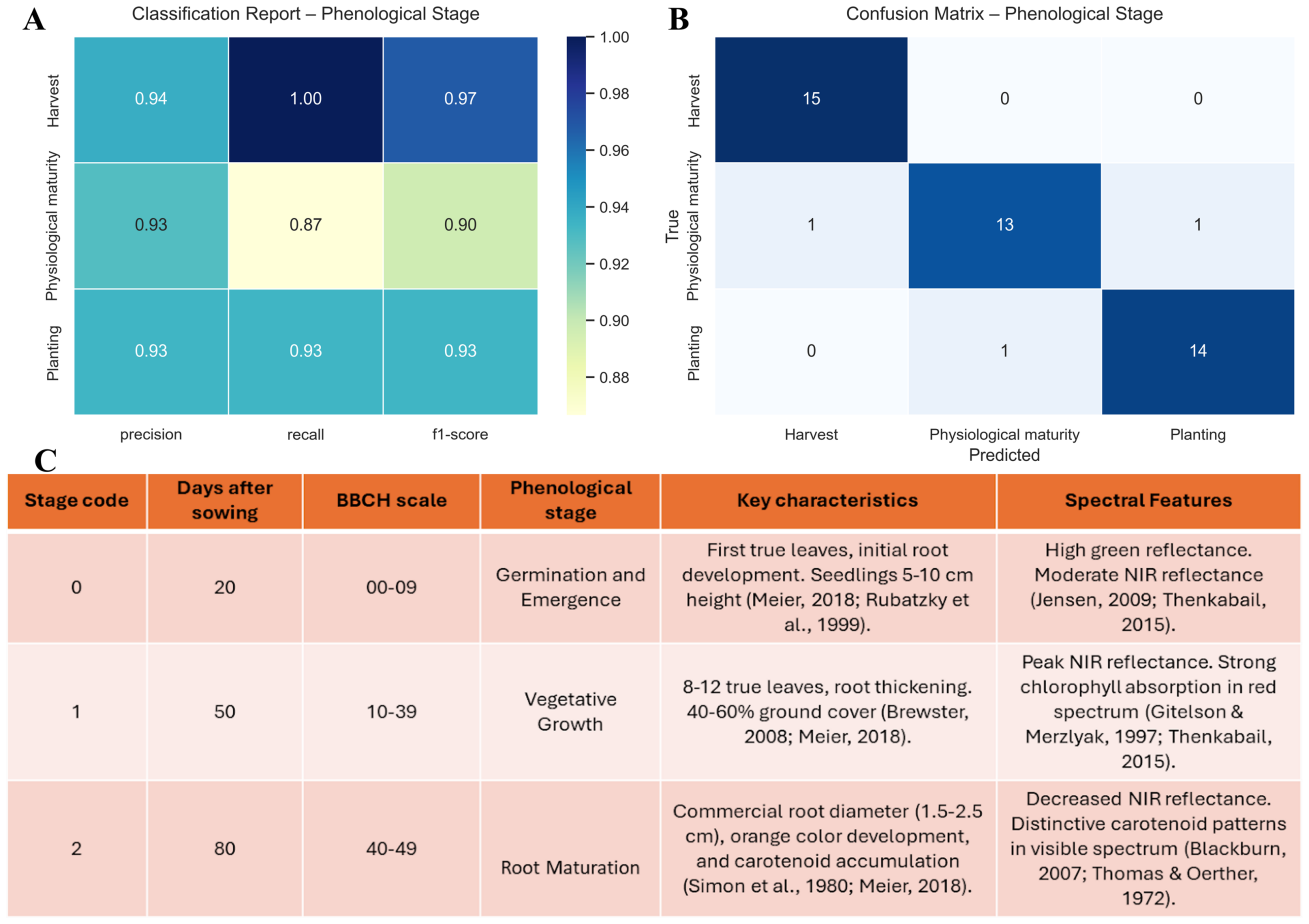
Performance evaluation of the phenological stage classification model. (A) Classification report summarizing precision, recall, and F1-score for the three phenological stages (Planting, Physiological maturity, and Harvest). (B) Confusion matrix showing correctly and incorrectly classified samples across stages. (C) Descriptive summary of phenological stages including BBCH scale, days after sowing, agronomic traits, and characteristic spectral responses. Results demonstrate high classification performance and clear spectral differentiation among developmental stages.

The classification model for identifying the crop’s phenological stage showed great accuracy, as summarized in the classification report in Fig. 4. The model achieved F1-scores above 0.90 for all classes (Planting: 0.93, Phenological maturity: 0.90, and Harvest: 0.97), demonstrating its ability to successfully distinguish between different developmental phases. This high classification capability is directly derived from the unique spectral and textural signature that each stage exhibits in the orthomosaics. The model learned to recognize the patterns described in the analysis. Planting Stage (Germination and Emergence) was characterized by its high green reflectance (due to sparse leaf cover and soil exposure) and NIR reflectance, as described in the table of Fig. 4. The initially heterogeneous and fragmented canopy texture also served as a key indicator for the model.

Vegetative Growth Stage was characterized by the highest NIR reflectance, resulting from a dense and healthy canopy structure, coupled with strong chlorophyll absorption in the red spectrum. The model associated this peak in “greenness” indices (such as NDVI and SAVI) and the more uniform, consolidated texture with the stage of peak vegetative vigor. The classification of harvest stage (Root Maturation) was the most accurate (F1-score of 0.97), driven by marked physiological changes. The model identified the combination of a decrease in NIR reflectance and chlorophyll indices (NDVI), along with a pronounced increase in the senescence index (PSRI). This spectral pattern, described in the table as “distinctive carotenoid patterns” and “decreased NIR reflectance,” provides an unambiguous signature of the transition towards maturation and the onset of senescence, allowing for a clear distinction from earlier stages.

#### Predictive carotenoid determination model based on multispectral imagen field data

**Figure 5:**
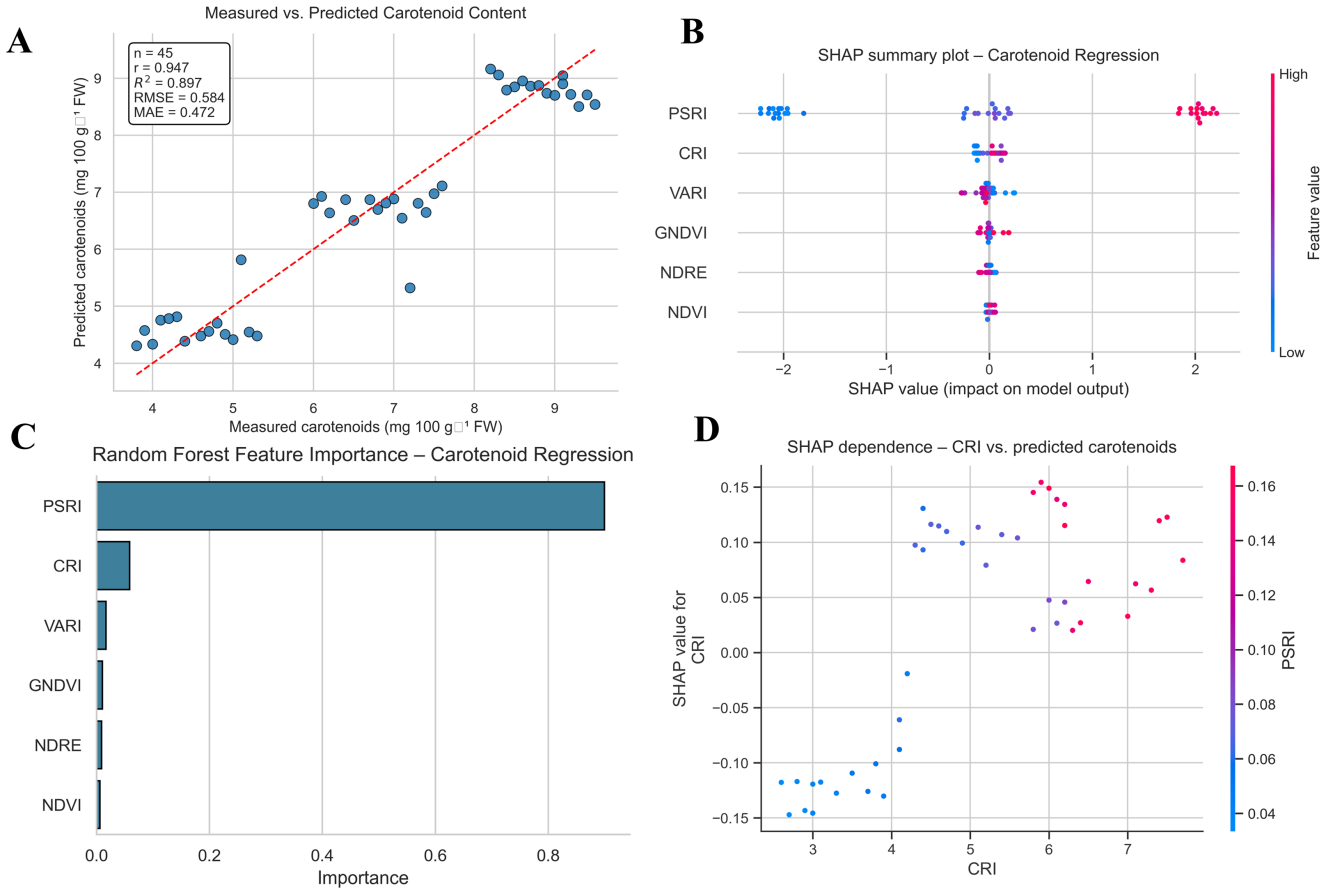
Predictive performance and interpretability of the carotenoid regression model based on spectral indices. (A) Relationship between measured and Random Forest predicted carotenoid concentration (mg 100 g^1^ FW). (B) SHAP summary plot indicating the relative contribution of spectral indices to model predictions. (C) Random Forest feature-importance ranking highlighting PSRI as the dominant predictor. (D) SHAP dependence plot showing the interaction between CRI and predicted carotenoid concentration. These analyses confirm both predictive accuracy and physiological interpretability of the regression model.

The regression model developed to predict carotenoid content (mg 100 g^−1^ fresh weight) demonstrated high performance and reliability. As shown in Fig. 5A, the model achieved high predictive accuracy, with a R^2^ of 0.897 and a RMSE of 0.584, indicating that it explains nearly 90% of the variability in the measured data. The close alignment between the measured and predicted values in the scatter plot validates the model’s robustness.

The integration of spectral indices extracted from the orthomosaics was key to this success. Both the feature importance analysis (Random Forest Feature Importance) and the SHAP analysis (SHAP summary plot) consistently identified the Plant Senescence Reflectance Index (PSRI) and the CRI as the two most important variables for the model.

The high importance of PSRI (Fig. 5C) is directly related to the phenological trajectory observed in orthomosaics. PSRI is a specific indicator of senescence. The lowest value of this indicator during the peak of photosynthetic activity (Vegetative Growth stage) and its pronounced increase in the final stage (Root Maturation), as detailed in the temporal analysis, mark the onset of functional pigment degradation. The machine learning model learned that as PSRI increases (indicating senescence), the carotenoid content in the root tends to stabilize or be affected by degradation processes, making this index a fundamental predictor of the physiological state linked to carotenoid accumulation.

The high importance of CRI (Fig. 5C) is intuitive, as this index is designed to directly estimate carotenoid abundance. The SHAP dependence plot for CRI (Fig. 5B reveals the non-linear nature of its relationship with the model’s prediction. Low to medium CRI values have a positive impact on the prediction (positive SHAP values), which physiologically correlates with the active accumulation of carotenoids during the Root Maturation phase. However, the relationship plateaus or reverses at very high values, which could reflect index saturation or the onset of carotenoid degradation concomitant with advanced senescence, a pattern also hinted at in the temporal trend of CRI1.

The other indices (VARI, GNDVI, NDRE, NDVI) also contributed to the model, but to a lesser extent. Their contribution is linked to the strong positive correlation observed between NDVI, SAVI, and CRI1 during the growth phase, confirming that photosynthetically active green biomass is a prerequisite for the subsequent accumulation of carotenoids in the root.

#### Determination of quality parameters (carotenoids) in different genotypes of carrots using hyperspectral data acquired with a spectroradiometer

**Figure 6:**
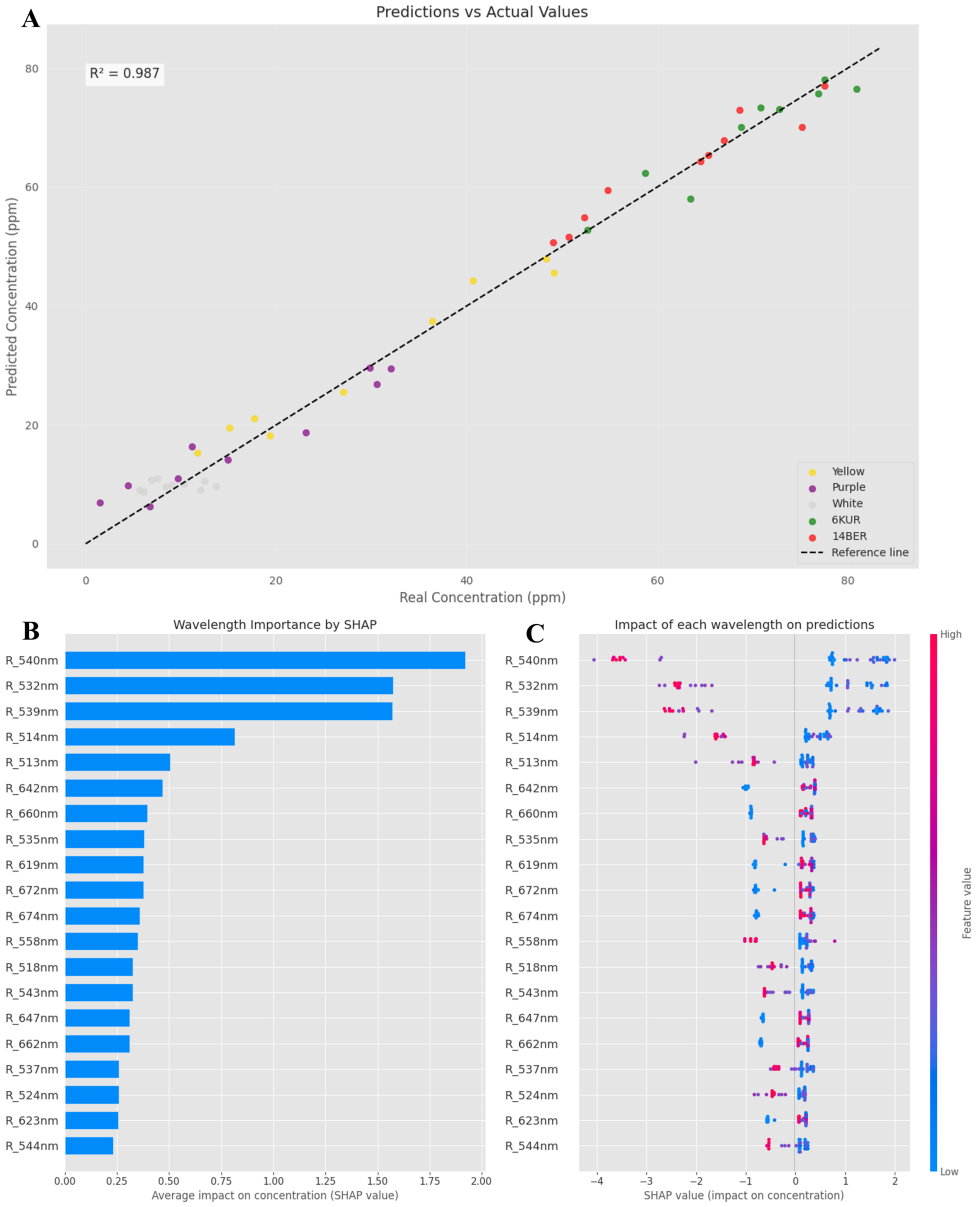
Hyperspectral regression model for carotenoid estimation using spectroradiometer signatures. (A) Observed *versus* predicted carotenoid concentration demonstrating strong model fit. (B) Wavelength importance ranked by mean SHAP value, identifying key spectral regions associated with carotenoid absorption. (C) SHAP dependence analysis illustrating the directional influence of each wavelength on prediction output. Results validate the robustness and biochemical relevance of the hyperspectral predictive model.

The predictive model developed to estimate carotenoid concentration directly from high-resolution spectral signatures demonstrated excellent performance. As shown in Fig. 6A, the model achieved a near-perfect fit with a coefficient of determination (R^2^) of 0.987. The minimal dispersion of data points around the 1:1 regression line confirms the model’s high precision and its ability to serve as an effective transfer function, converting spectral data into a reliable physiological metric.

The SHAP analysis (Fig. 6C) was critical for interpreting the model’s decision-making process in physiological terms. The analysis quantified the specific contribution of individual wavelengths, revealing which parts of the spectrum were most influential for the prediction. The SHAP analysis identified that increases in reflectance at 540 nm and 532 nm (Green Region) exerted the strongest positive influence on the predicted carotenoid concentration. Physiologically, this is a classic signature of carotenoid presence. Chlorophylls strongly absorb red and blue light, but carotenoids do not absorb strongly in the green region. Therefore, as carotenoid content increases relative to chlorophyll, reflectance in the green-yellow spectrum (around 550 nm) also increases. The model successfully learned this fundamental relationship: higher reflectance at 540 nm and 532 nm is a direct spectral indicator of higher carotenoid content and 700 nm is highly sensitive to chlorophyll content and plant stress. Its importance in the model suggests that the algorithm also leverages the physiological relationship between canopy chlorophyll status (which changes during senescence) and the concurrent accumulation or degradation of carotenoids in the root.

The inclusion of 514 nm and 513 nm in Blue-Green, albeit with lesser contributions, enhanced the model’s robustness. Reflectance in the blue–green region is influenced by the combined absorption of chlorophylls and carotenoids. Their inclusion likely helps the model fine-tune its predictions by accounting for the complex interplay between these pigments, improving generalizability across different physiological stages.

### Phase II. Determination of quality parameters (carotenoids) and damage using RGB images

#### Development of a colorimetric index using RGB images for the indirect determination of carotenoids

**Figure 7:**
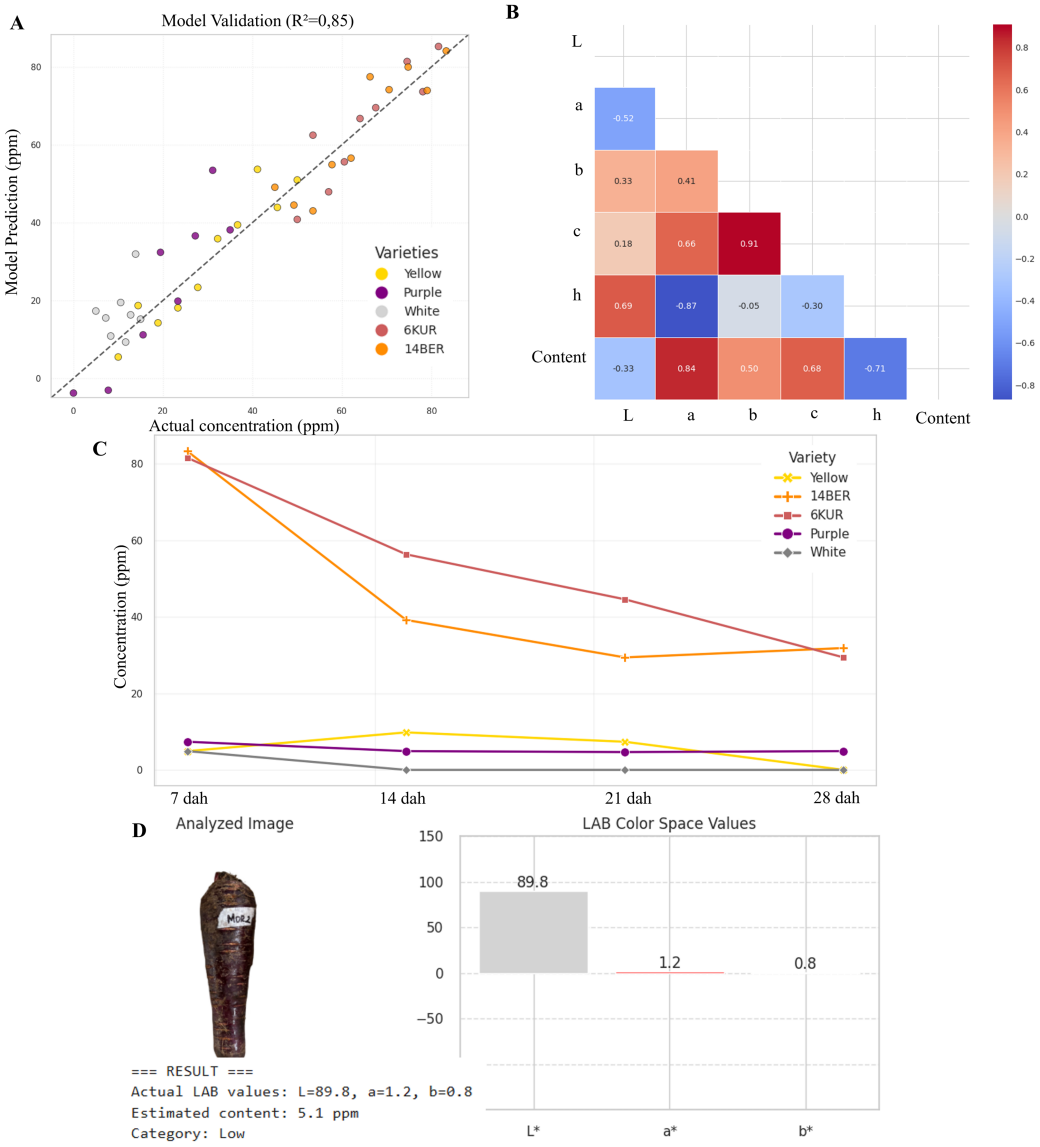
Validation of the ICarot index and colorimetric relationships with carotenoid content. (A) Scatter plot comparing predicted and reference carotenoid concentrations derived from colorimetric data. (B) Spearman correlation matrix between CIELAB color parameters and carotenoid concentration. (C) Temporal evolution of the ICarot index across carrot varieties during sampling dates. (D) Example of RGB-based image analysis showing LAB color extraction and estimated carotenoid content. Together, these results support the reliability of ICarot as a low-cost, non-destructive estimator of carotenoid concentration.

The predictive model for estimating total carotenoid content using RGB images demonstrated robust performance (Fig. 7A). Validation results showed a coefficient of determination (R^2^) of 0.85 between spectrophotometric reference values and image-based predictions, indicating that the model explains 85% of the variability in measured carotenoid concentrations. The predictions effectively covered the complete concentration range observed in the samples (0–80 ppm) and aligned closely with the ideal regression line (y=x). Error metrics further confirmed the model’s accuracy, with a RMSE of 4.2 and a MAE of 6.8 on the validation set.

Analysis of color space parameters revealed significant correlations with carotenoid content. The L* parameter (lightness) showed a strong negative correlation (−0.71) with carotenoid concentration, indicating that samples with higher pigment density appear darker. The b* parameter (yellow-blue component) demonstrated a moderate positive correlation (0.41), consistent with the yellow-orange coloration characteristic of carotenoids. The a* parameter (green-red component) showed a weaker but still meaningful positive correlation (0.33), suggesting some contribution from red tonalities to the overall pigment signature.

The temporal progression of the iCarot index (Fig. 7C) revealed a consistent pattern that strongly and inversely mirrored laboratory-measured carotenoid concentrations throughout the sampling period. This parallel evolution confirms the index’s reliability for monitoring pigment dynamics over time. When examining varietal differences, significant disparities in mean index values emerged (Kruskal–Wallis, *p* < 0.01), with the 14BER and 6KUR varieties exhibiting consistently higher iCarot values that correspond to their genetically determined greater carotenoid accumulation capacity.

Visual analysis of processed RGB images (Fig. 7D) provided practical validation of the model’s application. Examination of representative images showed clear correspondence between color patterns in the orange-yellow spectrum and the model’s predicted concentration maps. Automated pixel classification into nutritional categories based on established thresholds (“Low”: <20 ppm, “Medium”: 20–50 ppm, “High”: >50 ppm) consistently matched destructively measured values, demonstrating the model’s effectiveness for rapid, non-destructive carotenoid screening.

## Discussion

The results of this study validate the hypothesis that the integration of spectral and colorimetric techniques with artificial intelligence models enables the non-destructive estimation of carotenoid content, physicochemical and phenological parameters, and high-accuracy classification of damage in carrots. The apparent separation between the field-scale spectral monitoring and the postharvest colorimetric assessment reflects two complementary observational scales of the same physiological process. Phenological maturity, detected through vegetation indices and spectral dynamics, is intrinsically linked to carotenoid biosynthesis and accumulation within the storage root. Consequently, Phase I establishes the temporal and physiological context in which carotenoid content reaches its maximum, while Phase II provides a practical, image-based quantification of that biochemical state. This multi-scale structure was therefore intentionally designed to connect canopy-level maturity detection with root-level nutritional quality, ensuring biological coherence rather than representing two independent studies.

The predictive model based on the spectral signature achieved a R^2^ of 0.987, demonstrating a near-perfect correlation with the reference data. This finding aligns with Gitelson, Keydan & Merzlyak (2006), who highlighted the sensitivity of spectral bands in the 530–570 nm range for carotenoid quantification. Furthermore, the identification of 540 nm and 532 nm as the most determinant wavelengths reinforces previous evidence of the relationship between these spectral ranges and pigment concentration (Surjadinata, Jacobo-Velázquez & Cisneros-Zevallos, 2021). These results address a critical gap in the literature by providing a standardized method for pigmented varieties, which traditionally present challenges due to interference from other phenolic compounds, such as anthocyanins and chlorophylls (Montesano et al., 2020). These are naturally dark pigments that can mask the carotenoid signal (Montesano et al., 2020). Anthocyanins, which absorb strongly in the 500–600 nm range, and chlorophyll, with its absorption peaks in the blue and red regions, can spectrally overlap with carotenoids if specific bands are not selected, as was done in this study to isolate their signal (Gitelson, Keydan & Merzlyak, 2006).

The SHAP-based analysis provided not only statistical interpretability but also physiological insight into the spectral drivers of carotenoid prediction (Wu et al., 2025). The prominence of wavelengths located in the green and red-edge regions is consistent with well-established plant physiological processes associated with pigment accumulation and senescence dynamics (Noda, Muraoka & Nasahara, 2021). Variations in the red-edge region are strongly linked to chlorophyll degradation and structural canopy changes during maturation, which indirectly enhance the spectral detectability of carotenoids as chlorophyll masking decreases (Yu et al., 2025). Similarly, reflectance patterns in the green spectral region are sensitive to shifts in the balance between chlorophyll and carotenoid pigments, reflecting progressive carotenoid prominence during root development and physiological maturation (Lee et al., 2024). These relationships agree with previous studies describing spectral transitions associated with pigment composition and senescence in horticultural crops, supporting the biological plausibility of the machine-learning predictions. Consequently, the SHAP results indicate that the predictive models are not driven solely by statistical correlations but instead capture spectrally and physiologically meaningful signals, reinforcing the credibility and interpretability of the proposed artificial intelligence–based framework for non-destructive carotenoid assessment.

The development of the ICarot index, derived from standardized CIELAB parameters, provides a practical and accessible approach for the indirect estimation of carotenoid content, achieving strong predictive performance (*R*^2^ = 0.85). Compared with conventional colorimetric indices such as Chroma or Hue, ICarot demonstrates greater robustness to variations in illumination conditions and cultivar diversity, addressing known limitations of traditional color-based methods when applied to non-standard genotypes (Pathare, Opara & Al-Said, 2013). The strong negative correlation observed between the L* parameter and carotenoid concentration (−0.71) is consistent with previous findings linking higher lightness values to lower pigment accumulation (Wu & Sun, 2013). Importantly, this methodological advantage reduces dependence on costly analytical equipment, such as spectroradiometers, thereby facilitating broader adoption of nutritional quality monitoring in resource-limited agricultural systems.

From a scalability and industrial implementation perspective, the ICarot index represents a viable alternative to spectrally intensive approaches. Because it can be computed from images acquired with low-cost RGB cameras, ICarot challenges the assumption that high-cost instrumentation is a prerequisite for precision agriculture (Pathare, Opara & Al-Said, 2013). Its integration into postharvest sorting lines or simple computer vision systems enables automated product valorization based on nutritional quality, improving operational efficiency and reducing food waste by minimizing false rejection of healthy produce (Li et al., 2021). Moreover, this approach aligns with current trends toward data-driven, market-oriented production systems, supporting hyper-segmentation strategies that enhance traceability, optimize value chains, and increase profitability for both small-scale producers and agribusinesses (Wolfert et al., 2017; Lehmann, Reiche & Schiefer, 2012).

The theoretical foundation of this study is grounded in the well-established relationship between the optical properties of plant tissues and their underlying biochemical composition (Cen & He, 2007). Through integrating spectral indices sensitive to pigment dynamics (*e.g.*, CRI1 and PSRI) with texture metrics derived from grey-level co-occurrence matrices, the proposed framework captures not only carotenoid concentration but also their spatial distribution across plant tissues an aspect that remains underexplored in previous studies focused primarily on mean spectral responses (Thenkabail, Lyon & Huete, 2019). The strong temporal correspondence observed between the ICarot index and vegetative senescence further provides mechanistic insight into carotenoid dynamics and harvest optimization, a key objective in precision agriculture (Gamon et al., 2016). This relationship is biochemically supported by chlorophyll degradation during senescence, which reduces competitive light absorption and progressively reveals the more stable carotenoid pigments, leading to shifts in CIELAB parameters (lower L* values and changes in a* and b*) and consequently higher ICarot values (Hörtensteiner, 2009). Thus, ICarot reflects not only carotenoid accumulation but also physiological maturity and the onset of senescence.

While the developed models demonstrate high performance in estimating carotenoids and classifying phenological stages, it is essential to acknowledge certain limitations. First, the reliance on specific lighting conditions for capturing photographs and RGB images represents a significant challenge, as variations in light intensity, shadows, or changes in solar angle can affect the accuracy of derived colorimetric and spectral indices. Although training with orange, yellow, and purple varieties enabled the development of more robust models capable of isolating the carotenoid signal even in the presence of anthocyanins, generalizing to other genotypes and environmental conditions requires further validation across multiple locations and growing seasons. Additionally, transitioning these models to operational environments, such as packing lines or field monitoring, will necessitate the development of standardized lighting and calibration protocols, as well as the use of cost-effective sensors optimized for the critical wavelengths identified (*e.g.*, 540 nm). Finally, integrating destructive biochemical analyses in future studies will help solidify the relationship between the identified spectral patterns and underlying physiological mechanisms, thereby ensuring the scalability and applicability of these tools in commercial agricultural systems.

Despite the strong predictive performance achieved, several practical limitations must be acknowledged. RGB-based estimation depends on controlled illumination conditions, and variability in light intensity, shadows, or solar angle may influence the accuracy of derived colorimetric and spectral indices. Although the inclusion of multiple pigmented varieties improved robustness against anthocyanin interference, broader validation across genotypes, environments, and growing seasons is required to confirm generalizability. Operational deployment in field or industrial environments will require standardized calibration procedures, stable lighting systems, and cost-effective sensors optimized for the critical wavelengths identified (*e.g.*, approximately 540 nm). In addition, computational requirements associated with machine-learning inference and deployment may limit real-time implementation in low-resource settings without optimized hardware or cloud-based processing.

Nevertheless, the integration of low-cost RGB sensing and simplified spectral indicators such as ICarot represents a promising pathway toward economically accessible precision-agriculture tools, particularly for small- and medium-scale producers. Future research should therefore prioritize multi-environment validation, sensor miniaturization, automated calibration strategies, and real-time deployment pipelines to enable scalable adoption in commercial production and postharvest management systems while strengthening the causal linkage between spectral signatures and underlying physiological mechanisms.

## Conclusions

This study demonstrates the effectiveness of an integrated, multi-sensor framework for the non-destructive assessment of phenological stages, carotenoid content, and quality in carrots by combining spectral techniques, colorimetry, and artificial intelligence. The high predictive performance achieved by the spectroradiometer-based model (*R*^2^ = 0.987), together with the robustness and accessibility of the ICarot colorimetric index (*R*^2^ = 0.85) and the phenological classification model, confirms that accurate quality monitoring can be achieved without reliance on destructive or high-cost analytical methods. Overall, the proposed approach establishes a scalable foundation for real-time crop monitoring and postharvest quality control, contributing to improved production efficiency, reduced losses, and enhanced sustainability in carrot production systems.

##  Supplemental Information

10.7717/peerj.21389/supp-1 Supplemental Information 1 Multispectral vegetation indices used to determine the condition of 12NAN variety carrots

10.7717/peerj.21389/supp-2 Supplemental Information 2 Texture indices used to determine the condition of 12NAN variety carrots

10.7717/peerj.21389/supp-3 Supplemental Information 3 Geometric indices used to determine the condition of 12NAN variety carrots

10.7717/peerj.21389/supp-4 Supplemental Information 4 Orthomosaics of different indices associated with the spectral response of plots cultivated with zabnahotia at different phenological stages

## Abbreviations

AI: Artificial Intelligence
BBCH: Biologische Bundesanstalt, Bundessortenamt and Chemical Industry scale
C: Chroma (color saturation in CIELAB space)
CIELAB: Commission Internationale de l’Éclairage Lab* color space
CRI: Carotenoid Reflectance Index
CRI1: Carotenoid Reflectance Index 1
DAS: Days After Sowing
*D. carota*: *Daucus carota*
GLCM: Grey-Level Co-Occurrence Matrix
GNDVI: Green Normalized Difference Vegetation Index
HPLC: High-Performance Liquid Chromatography
ICarot: Carotenoid Colorimetric Index
MAE: Mean Absolute Error
NDRE: Normalized Difference Red-Edge Index
NDVI: Normalized Difference Vegetation Index
NIR: Near-Infrared
ppm: Parts Per Million
PSRI: Plant Senescence Reflectance Index
RF: Random Forest
RMSE: Root Mean Square Error
RGB: Red, Green, Blue
R^2^: Coefficient of Determination
SAVI: Soil Adjusted Vegetation Index
SG: Savitzky–Golay filter
SMOTE: Synthetic Minority Over-sampling Technique
SNV: Standard Normal Variate
TSS: Total Soluble Solids
TTA: Total Titratable Acidity
UAV: Uncrewed Aerial Vehicle
VARI: Visible Atmospherically Resistant Index
VIS–NIR: Visible and Near-Infrared spectral region

## Additional Information and Declarations

### Data Availability

The following information was supplied regarding data availability: The data is available at GitHub and Zenodo: - https://github.com/agrocompuepidemlab/Carrot-value-chain-proyect/tree/main
- Ospina Sanchez, P. A. (2025). Carrot-value-chain-proyect [Data set]. Zenodo. https://doi.org/10.5281/zenodo.17872167.
