# Supplementary material for: Multi-scale predictive modeling of phenology and carotenoid content in carrots using spectral techniques, colorimetry, and artificial intelligence
Source: PeerJ. 2026 Jun 26;14:e21389. doi: 10.7717/peerj.21389 (PMC13312970; doi:10.7717/peerj.21389)
Supplement: Supplemental Information 1 [file peerj-14-21389-s001.docx]

| **Index** | **Equation** | **Function** | **Reference** |
| --- | --- | --- | --- |
| Normalized Difference Vegetation Index (NDVI) | $\frac{R_{NIR}-R_{RED}}{R_{NIR}+R_{RED}}$ | Sensitive to biomass and water stress. | Rouse et al. (1973) |
| Enhanced Vegetation Index (EVI) | $2.5\times\frac{R_{NIR}-R_{RED}}{R_{NIR}+6R_{RED}-7.5R_{BLUE}+1}$ | Reduces atmospheric and soil effects. | Huete et al. (1997) |
| Soil-adjusted vegetation index (SAVI) | $\frac{R_{NIR}-R_{RED}}{R_{NIR}+R_{RED}+L}\times(1+L)$ | Ideal for areas with sparse vegetation cover. | Huete et al. (1988) |
| Modified Soil-Adjusted Vegetation Index (MSAVI) | $\frac{2R_{NIR}+1\sqrt{{(2R_{NIR}+1)}^{2}-8R_{NIR}-R_{RED}}}{2}$ | Optimized for variations in vegetation cover | Qi et al., (1994) |
| Green Normalized Difference Vegetation Index (GNDVI) | $\frac{R_{NIR}-R_{GREEN}}{R_{NIR}+R_{GREEN}}$ | Sensitive to chlorophyll | Gao et al., (2024) |
| Carotenoid Reflectance Index 1 (CRI1) | $\frac{1}{R_{510}}-\frac{1}{R_{550}}$ | Designed to estimate carotenoid content | Gitelson et al., (2002) |
| Plant Senescence Reflectance Index (PSRI) | $\frac{R_{680}-R_{500}}{R_{750}}$ | Leaf senescence indicator | Merzlyak et al., (1999) |
| Structural Pigment Index (SIPI) | $\frac{R_{800}-R_{445}}{R_{750}}$ | Useful for assessing oxidative stress | Peñuelas et al. (1997) |
| Chlorophyll Vegetation Index (CVI) | $\frac{R_{NIR}}{R_{GREEN}}\times\frac{R_{RED}}{R_{GREEN}}$ | Related to chlorophyll concentration | Yan et al., (2025) |
| Normalized Difference Water Index (NDWI) | $\frac{R_{GREEN}-R_{NIR}}{R_{GREEN}+R_{NIR}}$ | Detects water content in leaves | Gao.(1996) |
