# Supplementary material for: Multi-scale predictive modeling of phenology and carotenoid content in carrots using spectral techniques, colorimetry, and artificial intelligence
Source: PeerJ. 2026 Jun 26;14:e21389. doi: 10.7717/peerj.21389 (PMC13312970; doi:10.7717/peerj.21389)
Supplement: Supplemental Information 2 [file peerj-14-21389-s002.docx]

| **Index** | **Equation** | **Function** | **Reference** |
| --- | --- | --- | --- |
| Contrast | $\sum_{i,j} \left\vert i-j \right\vert^{2}*p(i,j)$  ​ | Measures local variations in intensity | Haralick et al. (1973) |
| Dissimilarity | $\sum_{i,j} \left\vert i-j \right\vert*p(i,j)$ | Similar to contrast but less sensitive to sudden changes | Haralick et al. (1973) |
| Homogeneity | $\sum_{i,j} \frac{p(i,j)}{1+\left\vert i-j \right\vert}$ | Evaluates uniformity in intensity distribution | Haralick et al. (1973) |
| Energy (ASM - Second Angular Momentum) | $\sum_{i,j} {p\left( i,j \right)}^{2}$  ​ | Measures texture uniformity | Haralick et al. (1973) |
| Correlation | $\sum_{i,j} {\frac{\left( i-\mu_{i} \right)\left( j-\mu_{j} \right)*p\left( i,j \right)}{\sigma_{i}\sigma_{j}}}$ | Indicates linear dependence between neighboring pixels | Haralick et al. (1973) |
| Entropy | $-\sum_{i,j} {p\left( i,j \right)*{log}_{2}p\left( i,j \right)}$ | Quantifies randomness in the distribution of intensities | Haralick et al. (1973) |

#### 
