# Supplementary material for: Multi-scale predictive modeling of phenology and carotenoid content in carrots using spectral techniques, colorimetry, and artificial intelligence
Source: PeerJ. 2026 Jun 26;14:e21389. doi: 10.7717/peerj.21389 (PMC13312970; doi:10.7717/peerj.21389)
Supplement: Supplemental Information 3 [file peerj-14-21389-s003.docx]

| **Geometric Index** | **Definition** |
| --- | --- |
| Vegetation area | Total number of pixels classified as vegetation |
| Perimeter | Length of the edge of the vegetative region |
| Eccentricity | Ratio between the major and minor axes of the adjusted ellipsoid |
| Solidity | Ratio between the area and the convex area |
| Circularity | Degree of similarity to a perfect circle |
| Major and minor axes | Length (in pixels) of the axes of the fitted ellipse. |
| Orientation | Angle (in radians) of the major axis relative to the horizontal. |
| Convex area | Area of the minimum convex polygon that encompasses the region |
| Equivalent diameter | Diameter of a circle with equal area |
| Extension | Ratio between the area and the area of the bounding rectangle. |
| Feret diameter | Maximum distance between two points on the contour |

#### 
