# Supplementary figures and images for: Multi-scale predictive modeling of phenology and carotenoid content in carrots using spectral techniques, colorimetry, and artificial intelligence
Source: PeerJ. 2026 Jun 26;14:e21389. doi: 10.7717/peerj.21389 (PMC13312970; doi:10.7717/peerj.21389)

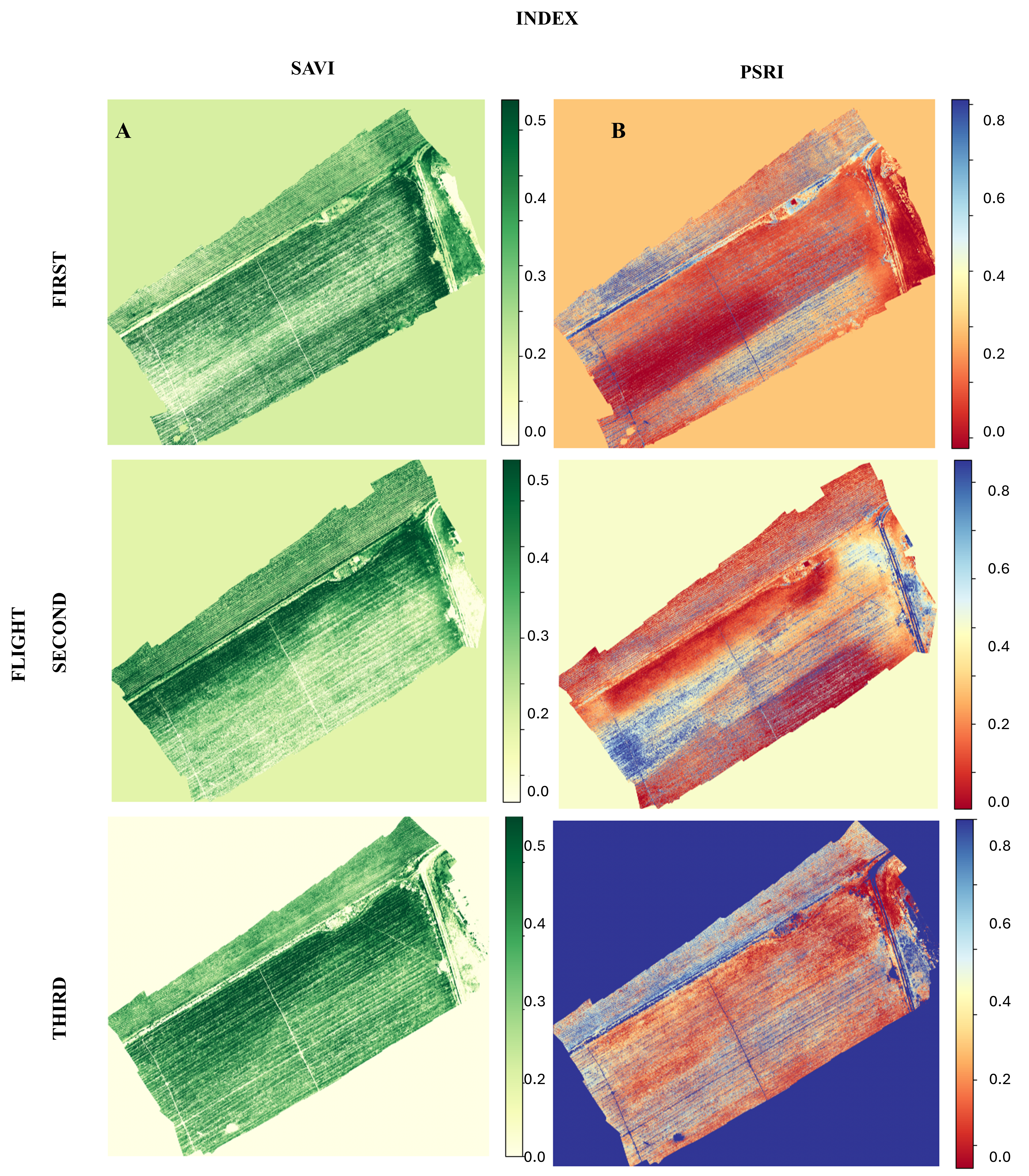

Supplement: Supplemental Information 4 [file peerj-14-21389-s004.png]
